# GASTROINTESTINAL COMPLICATIONS OF CORONAVIRUS DISEASE (COVID-19)

**DOI:** 10.1590/0102-672020210002e1620

**Published:** 2021-12-17

**Authors:** Marcelo Augusto Fontenelle RIBEIRO-JUNIOR, Samara de Souza AUGUSTO, Yasmin Garcia Batista ELIAS, Cássia Tiemi Kawase COSTA, Paola Rezende NÉDER

**Affiliations:** 1 Disciplina de Cirurgia Geral e do Trauma, Pontifícia Universidade Católica de São Paulo, Sorocaba, SP, Brasil; 2 Laboratório de Transplante e Cirurgia do Fígado, Faculdade de Medicina, Universidade de São Paulo, São Paulo, SP, Brasil; 3 Faculdade de Ciências Médicas de São José dos Campos Humanitas, Medicina; 4 Universida de Santo Amaro, São Paulo, SP, Brasil

**Keywords:** Coronavirus infections, SARS-CoV, Gastrointestinal tract, Hemorrhage, Ischemia, Pancreatitis, Infecções por coronavirus, SARS-CoV, Trato gastrointestinal, Hemorragia, Isquemia, Pancreatite

## Abstract

***Background*:**

It is currently understood that severe acute respiratory syndrome coronavirus-2 (SARS-CoV-2) directly enters target cells by binding to the angiotensin-converting enzyme 2 (ACE2) receptor. Accordingly, tissues with high expression levels of ACE2 are more susceptible to infection, including pulmonary alveolar epithelial cells, small intestine enterocytes, cholangiocytes, and vascular endothelial cells. Considering the atypical manifestations of COVID-19 and the challenges of early diagnosis, this review addresses the possible gastrointestinal complications of the disease.

**Method:**

The phrase “Gastrointestinal complication of COVID” was searched in the PubMed, Medline, and SciELO databases. Due to the heterogeneity of the studies included in the present review, a narrative synthesis of the available qualitative data was performed.

**Result:**

The literature search retrieved 28 articles, primarily case reports and case series, for the qualitative analysis of gastrointestinal complications of COVID-19, in addition to two retrospective cohort and one case-control. The studies focused on hemorrhagic, thrombotic, ischemic, and perforation complications, in addition to acute pancreatitis and pneumatosis intestinalis.

**Conclusion:**

There is a straight relationship between high expression levels of ACE2 in the gastrointestinal tract and its greater susceptibility to direct infection by SARS-CoV-2. So, it is important to consider the gastrointestinal infection manifestations for early diagnosis and treatment trying to avoid more serious complications and death.

## RESUMO

**Racional:**

Atualmente, entende-se que o vírus SARS-CoV-2 é capaz de infectar diretamente células-alvo por acoplamento ao receptor da enzima conversora de angiotensina 2 (ECA 2), por isso tecidos que contêm altos níveis de ECA 2 estão mais suscetíveis a infecção, como as células epiteliais dos alvéolos pulmonares, os enterócitos do intestino delgado, os colangiócitos e o endotélio vascular.

**Objetivo:**

Levando em consideração as manifestações atípicas da COVID-19 e a dificuldade na suspeita diagnósticaprecoce, esta revisão busca apresentar aspossíveis complicações gastrointestinais da doença.

**Método:**

A busca foi realizada com o descritor“Gastrointestinal complication of COVID” nas bases de dados PubMed, Medline e SciELO e dada à natureza heterogênea dos textos adicionados a esta revisão, optou-se pela realização uma síntese qualitativa narrativa dos dados disponíveis.Foram selecionados 28 artigos para análise qualitativa sobre o tema, sendo principalmente relatos e séries de casos, além de duas coortes retrospectivas e um caso-controle.Os estudos tratavam de complicações hemorrágicas, trombóticas, isquêmicas, perfurativas, além de pancreatite aguda e pneumatose intestinal.

**Resultados:**

Diversos autores sugerem que o vírus tenha um papel direto no dano às células da mucosa gastrointestinal. Os estudos relatam casos de hemorragia gastrointestinal, assim como fenômenos trombóticos e isquêmicos, reforçando o papel do SARS-CoV-2 nos distúrbios de coagulação. Casos de perfuração intestinal e pancreatite também foram associados à COVID-19.

**Conclusão:**

Uma vez que a relação entre altos níveis de ECA 2 no trato gastrointestinal e a sua maior suscetibilidade a infecção direta pelo vírus SARS-CoV-2 esteja estabelecida, é importante estar atento para as diferentes manifestações e complicações gastrointestinais e serem elas diagnosticadas e tratadas precocemente.

## INTRODUCTION

Cases of viral pneumonia associated with a severe acute respiratory syndrome were reported in December 2019 in the city of Wuhan, China. A novel coronavirus (severe acute respiratory syndrome coronavirus 2 [SARS-CoV-2]), the causative agent of coronavirus disease 2019 (COVID-19), was identified in January 2020^39^ ^,^ ^43^ ^,^ ^48^. After rapidly spreading across continents, the World Health Organization declared a public health emergency of international concern, described as a pandemic, on March 11, 2020^47^. As of early January 2021, Johns Hopkins University (Baltimore, MD, USA) confirmed 92,240,036 cases globally, of which 1,975,707 were fatal^17^.

Since September 2020, the world has witnessed a “second wave” of COVID-19, associated not only with an increase in the number of cases, but also with the emergence of new SARS-CoV-2 variants^12^ ^,^ ^15^. 

The primary symptoms of COVID-19 include fever, dry cough, dyspnea, fatigue, myalgia, and headache^45^. Although poorly related to the disease, extrapulmonary symptoms have also been documented since the beginning of the pandemic, with gastrointestinal symptoms being the most relevant. Some patients experience gastrointestinal manifestations in the early stages of the disease, including nausea, vomiting, diarrhea, abdominal pain, and anorexia^8^ ^,^ ^28^ ^,^ ^31^ ^,^ ^37^ ^,^ ^45^. While others progress without exhibiting any respiratory symptoms^16^ ^,^ ^20^ ^,^ ^41^.

It is currently understood that SARS-CoV-2 can directly infect target cells by binding to the angiotensin-converting enzyme 2 (ACE2) receptor. Accordingly, tissues with high expression levels of this receptor are more susceptible to infection. Tissues with higher receptor concentrations include pulmonary alveolar epithelial cells, small intestine enterocytes, cholangiocytes, and vascular endothelial cells^1^ ^,^ ^8^ ^,^ ^18^ ^,^ ^20^ ^,^ ^31^ ^,^ ^38^ ^,^ ^46^. ACE2 acts to regulate the inflammatory response, and its distribution in abdominal organs possibly explains the extrapulmonary symptoms experienced by some patients. Cellular alteration(s) result in intestinal and hepatic inflammation, as well as alteration(s) to the intestinal microbiota^1^ ^,^ ^21^.

Furthermore, gastrointestinal and respiratory comorbidities can increase the severity of the disease. However, gastrointestinal symptoms can be underdiagnosed because they usually manifest in the early stages of disease and are self-limited, thus making it difficult to correlate them with a diagnosis of COVID-19^20^.

Considering the atypical manifestations and challenges of early diagnosis, this review addresses possible gastrointestinal complications within the current context of the COVID-19 pandemic.

## METHODS

### Search strategy

A literature search of the PubMed, Medline, and SciELO databases was performed in November 2020 using the phrase “Gastrointestinal complication of COVID”. The search was limited to articles published in English and Spanish, and filters for type of study or date were initially not used.

### Screening and evidence synthesis

The literature search retrieved 587 articles. After excluding 248 duplicate articles, 339 were analyzed with regard to title and abstract, with six excluded for incompatible dates (published before 2020) and 227 for focusing on other topics. Of the remaining 106 articles, 81 were excluded for not addressing complications, although they focused on gastrointestinal involvement in COVID-19. After a manual search of citations, an additional four articles were included for relevance. 

**FIGURE 1:**
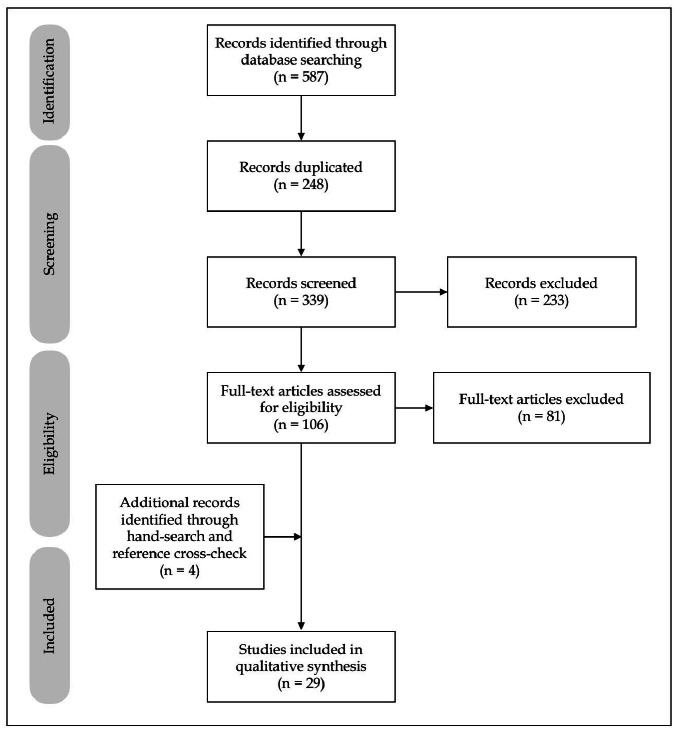
PRISMA flow diagram for article identification and sorting.

Ultimately, 29 articles were included in the present review (Figure 1).

### Statistical analysis

Due to the heterogeneous nature of the studies included in this review, a narrative synthesis of the qualitative available data was performed.

## RESULTS

A total of 28 articles, primarily case reports and case series, were selected for a narrative synthesis of qualitative data addressing the gastrointestinal complications of COVID-19, in addition to two retrospective cohort studies and one case-control. The studies reported on hemorrhagic, thrombotic, ischemic, and perforation complications, in addition to acute pancreatitis and pneumatosis intestinalis^3^ ^-^ ^7^ ^,^ ^9^ ^-^ ^11^ ^,^ ^13^ ^,^ ^14^ ^,^ ^18^ ^,^ ^19^ ^,^ ^22^ ^-^ ^27^ ^,^ ^29^ ^,^ ^30^ ^,^ ^32^ ^-^ ^36^ ^,^ ^40^ ^,^ ^42^ ^,^ ^44^. (Tables 1 to 5)

**TABLE 1:** Gastrointestinal hemorrhage

Title	Author	Study type	Complications	n	Outcome
A severe coronavirus disease 2019 patient with high-risk predisposing factors died from massive gastrointestinal bleeding: a case report	Chen et al.^7^	Case report	Massive gastrointestinal bleeding	1	Death (n=1)
Haemorrhagic enteritis and COVID-19: causality or coincidence	Amarapurk et al.^3^	Case report	Haemorrhagic enteritis	1	Discharge (n=1)
Peptic ulcer disease as a common cause of bleeding in patients with coronavirus disease 2019	Melazzini et al.^35^	Case report	Gastrointestinal bleeding from peptic ulcer	5	Death (n=1) Discharge (n=4)
Uncommon presentation of COVID-19: gastrointestinal bleeding	Gulen et al.^22^	Case report	Gastrointestinal bleeding	1	Discharge (n=1)
Upper gastrointestinal bleeding caused by SARS-CoV-2 infection	Li et al.^29^	Case report	Esophagus bleeding	1	Death (n=1)
Upper gastrointestinal bleeding in COVID-19 inpatients: incidence and management in a multicenter experience from Nothern Italy	Mauro et al.^3^ ^3^	Retrospective cohort	Upper gastrointestinal bleeding	23	Discharge (n=18) Death (n=5)
Clincal and intestinal histpatological findings in SARS-CoV-2/ COVID-19 patients with hematochezia	Cho et al.^11^	Case report	Low gastrointestinal bleeding	2	Discharge (n=2)
Self-limited gastrointestinal bleeding in COVID-19	Barret et al.^4^	Case series	Gastrointestinal bleeding	6	Spontaneous resolution (n=6)
Gastrointestinal bleeding in patients with coronavirus disease 2019: a matched case-control study	Martin et al.^32^	Retrospective, multicenter, matched 1:2 case-control	Gastrointestinal bleeding	41	No need for intervention (n=34) Intervention with hemostasis (n=7)
Gastrointestinal bleeding in patients with severe SARS-CoV-2	Gadiparthi et al.^19^	Case series	Gastrointestinal bleeding	3	Solved (n=2) Recurrent (n=1)

**TABLE 2:** Thrombosis and ischemia

Title	Author	Study type	Complications	n	Outcome
Superior mesenteric artery thrombosis and acute intestinal ischemia as a consequence of COVID-19 Case report infection	Cheung et al.^10^	Case report	Superior mesenteric artery thrombosis and acute intestinal ischemia	1	Discharge (n=1)
Perforated acute abdomen in a patient with COVID-19: an atypical manifestation of the disease	Corrêa Neto et al.^13^	Case report	Gastrointestinal ischemia	1	Death (n=1)
Gastrointestinal complications in critically ill patients with COVID-19	Kaafarani et al.^18^	Case series	Paralytic ileum; Ogilvie-like syndrome; hepatic injury; intestinal ischemia	141	Death (n=21)
Coronavirus disease 2019 (COVID-19) and ischemic colitis: an under-recognized complication	Chan et al.^6^	Case report	Ischemic colitis	1	Death (n=1)
Acute mesenteric thrombosis in two patients with COVID-19. Two cases report and literature review	Rodriguez-Nakamura et al.^40^	Case report and literature review	Mesenteric thrombosis	2	Discharge (n=1) Death (n=1)
A case report on spontaneous hemoperitoneum in COVID-19 patient	Karki et al.^26^	Case report	Splenic infarct and hemoperitoneum	1	Spontaneous resolution (n=1)

**TABLE 3:** Perforation

Title	Author	Study type	Complications	n	Outcome
Bowel perforation in a COVID-19 patient: case report	De Nardi et al.^14^	Case report	Right colon perforation	1	Discharge (n=1)
Paralytic ileus: a potential extrapullmonary manifestation of severe COVID-19	Ibrahim et al.^24^	Case report	Paralytic ileus and colon perforation	2	Recovering (n=1) Dialysis (n=1)
Gastrointestinal perforation in a critically ill patient with COVID-19 pneumonia	Dick et al.^25^	Case report	Gastric or duodenal ulcer perforation	1	Death (n=1)
Intestinal perforation caused by COVID-19	Nahas et al.^3^ ^6^	Case report	Left cólon perforation	1	Death (n=1)

**TABLE 4:** Acute pancreatitis

Title	Author	Study type	Complications	n	Outcome
SARS-CoV RNA detection in a pancreatic pseudocyst sample	Schepis et al.^4^ ^2^	Case report	Pancreatic pseudocyst	1	-
COVID-19 presenting as acute pancreatitis	Aloysius et al.^2^	Case report	Acute pancreatitis	1	Discharge (n=1)
Recurrent acute pancreatitis in a patient with COVID-19 infection	Cheung et al.^9^	Case report	Recurrent acute pancreatitis	1	Recurrent (n=1)
Case report: novel coronavirus - potential cause of acute pancreatitis?	Bokhari et al.^5^	Case report	Acute pancreatitis	1	Discharge (n=1)
Coronavirus disease-19 (COVID-19) associated with acute necrotizing pancreatitis (ANP)	Kumaran et al.^27^	Case report	Acute necrotizing pancreatitis	1	Recovering (n=1)
Coronavirus disease-19 (COVID-19) associated with severe acute pancreatitis: case report on three family members	Hadi et al.^23^	Case report	Acute pancreatitis	3	Intensive care unit (n=2) Death (n=1)
Pancreatic injury patterns in patients with COVID-19 pneumonia	Wang et al.^44^	Case series	Acute pancreatitis; hepatic injury	52	-
ACE2 expression in pancreas may cause pancreatic damage after SARS-CoV-2 infection	Liu et al.^30^	Cohort	Acute pancreatitis	121	-

**TABLE 5:** Pneumatosis intestinalis

Title	Author	Study type	Complications	n	Outcome
Pneumatosis intestinalis in COVID-19	Meine et al.^34^	Case report	Pneumatosis intestinalis	1	Discharge (n=1)

## DISCUSSION

### Gastrointestinal hemorrhage

Considering the atypical manifestations of COVID-19, primary care for patients experiencing extrapulmonary symptoms can be initiated without early diagnosis to avoid more serious complications when associated with respiratory conditions. Although gastrointestinal symptoms often present early and tend to evolve in the most severe forms of the disease, they are the only symptoms of the disease in some cases^20^.

A case presented by Amarapurkar et al.^3^ described a patient with an initial diagnostic hypothesis of acute abdomen due to hemorrhagic enteritis. On admission, the patient presented with intense abdominal pain and vomiting, which were indications for abdominal tomography. Nonspecific findings of the examination, including a large segment of the small bowel with a thickened wall, probably indicating hemorrhage with mild ascites, prompted exploratory laparotomy with small bowel resection and ileostomy. Histopathological analysis revealed extensive transmural hemorrhage, with the presence of fibrin inside the capillaries. In the postoperative period, real-time polymerase chain reaction (RT-PCR) testing for SARS-CoV-2 was positive, without other classic symptoms of COVID-19. In this case, the patient exhibited only extrapulmonary symptoms, leading to the hypothesis that gastrointestinal symptoms were present regardless of the absence of respiratory symptoms.

Because there are similar progressions of the disease, several authors have suggested that SARS-CoV-2 has a direct role in damage to cells of the gastrointestinal mucosa^4^ ^,^ ^7^ ^,^ ^20^ ^,^ ^22^ ^,^ ^32^ ^,^ ^35^. Gastrointestinal hemorrhage is one of the most common extrapulmonary complications and can occur due to primary or secondary causes: direct invasion by the virus or tissue hypoxia due to existing coagulopathy in patients who develop the disease^22^.

A retrospective study by Mauro et al.^33^ assessed patients with signs of upper gastrointestinal hemorrhage and tested positive for COVID-19. The mean age of the patients was 75 years and, after performing upper gastrointestinal endoscopy in some, peptic ulcer was the most common finding (44%), followed by hemorrhagic gastritis (22%). Of the 23 patients, 18 were discharged and five died due to worsening of the infection, corresponding to a mortality rate of 21.7%.

As Mauro et al.^33^, a multicenter retrospective case-control (1:2) study reported that gastrointestinal hemorrhage was found, on average, in 2% to 13% of patients with COVID-19. Those with gastrointestinal hemorrhage were paired with controls with equal clinical status and severity of COVID-19. Of these, 80% experienced upper gastrointestinal hemorrhage, with a predominance of gastric or duodenal ulcers and esophagitis, and 50% experienced lower gastrointestinal hemorrhage, mainly due to rectal ulcers^3^ ^2^.

A case series investigating SARS-CoV-2 infection by Barrett et al.^4^ reported that six patients who experienced gastrointestinal hemorrhage demonstrated an increased risk for bleeding among those with COVID-19. Of these patients, ranging in age from 66-77 years, all had ≥1 comorbidities. Hemorrhage was due to hematochezia (n=2), melena (n=2), ischemia of the left lower limb (n=1), and dyspnea only (n=1). Of all patients, four concomitantly presented the most common symptoms of COVID-19. The authors suggested that direct action of the virus led to mucosal damage and the development of gastrointestinal hemorrhage.

A case report by Li et al.^29^ demonstrated that it is possible to confirm the hypothesis of direct damage to the intestinal epithelium. A 77-year-old male with typical symptoms of COVID-19 was diagnosed with the disease. Among 98 hospitalized patients, he was the only one to evolve with coffee ground vomitus, indicating upper gastrointestinal hemorrhage. Upper endoscopy revealed herpetic lesions and superficial ulcers in which biopsy confirmed SARS-CoV-2 RNA. Direct infection by the virus in the esophagus was confirmed, thus supporting the hypothesis that ACE2 is the access route to gastrointestinal cells.

It is currently known that epithelial cells in the gastrointestinal tract express ACE2 - the receptor by which SARS-CoV-2 gains entry -; however, it remains controversial whether the virus can cause direct damage to the gastrointestinal epithelium and lead to ulceration and hemorrhage^4^ ^,^ ^7^ ^,^ ^19^ ^,^ ^32^. Another possible cause of gastrointestinal hemorrhage is coagulopathy, which is prevalent in patients with COVID-19 due to the state of hypercoagulability associated with pathophysiological processes of the disease^11^. As a result, treatment includes prophylactic anticoagulation, thus increasing the risk for gastrointestinal hemorrhage associated with the disease^4^.

### Thrombosis and ischemia

Coagulation dysfunction is one of the main causes of death in patients with severe COVID-19, who are more likely to exhibit a state of hypercoagulability, with manifestations of intravascular coagulation due to local damage^10^ ^,^ ^13^. Coagulopathy, high levels of D-dimer, and fibrinogen at the time of hospital admission for COVID-19 have been associated with a poorer clinical course, and higher risk for micro- and macro-circulatory thrombosis, and a higher mortality rate in hospitalized patients^6^ ^,^ ^10^.

The absence of important predisposing factors for thromboembolic formation in a patient who developed thrombosis in the superior mesenteric artery and acute intestinal ischemia suggested and reinforced the theory that there is a causal relationship between COVID-19 and hypercoagulability^10^. In a case reported by Correa Neto et al.^1^ ^3^, the patient exhibited increased D-dimer levels and a positive PCR test for COVID-19, in addition to a computed tomography scan revealing pneumothorax and extensive pneumoperitoneum, with the latter caused by ischemia of the entire gastrointestinal tract followed by colon perforation. The authors believed that the septic and thromboembolic events that precipitated the clinical condition was caused - directly or indirectly - by viral infection.

Corroborating these studies, the authors of a case report describing ischemic colitis manifesting as episodes of bloody diarrhea reaffirm the hypothesis of ACE2 having a direct role in infection of the gastrointestinal tract and describe the state of hypercoagulability promoted by the virus as one of the main risk factors for the complication^6^. In cases reported by Rodriguez-Nakamura et al.^40^, two patients with COVID-19 developed acute mesenteric thrombosis, with one experiencing hemoperitoneum and intestinal necrosis, while the other exhibited thrombosis of the mesenteric and portal veins, thus emphasizing the importance of considering thrombotic complications in COVID-19 patients.

A 32-year-old patient with no known comorbidities experienced splenic infarction and laceration, and intraperitoneal collection in the perisplenic, perihepatic, and pelvic cavities, with spontaneous resolution. The authors discussed evidence of common thromboembolic events in COVID-19, probably resulting from viral infiltration of the endothelium, with immune reaction and lymphocytic infiltration, leading to leukocytoclastic vasculitis and small vessel hyperplasia, with thickening and stenosis, which can lead to visceral infarction, as in the case described^26^. Kaafarani et al.^18^ analyzed 141 COVID-19 patients with gastrointestinal complications, of whom four experienced severe ileus and exhibited clinical and radiological features related to intestinal ischemia, requiring emergency surgery and intestinal resection. Small vessel thrombosis induced by SARS-CoV-2 or viral enteric neuropathy are two possible hypotheses that warrant further investigation.

The management of these patients should include coagulation tests, including coagulation profile, platelet count and D-dimer levels, to help determine a prognosis and dosages of anticoagulant agents, and imaging studies for early diagnosis to increase the chances for survival^10^ ^,^ ^40^. It is important to note that thrombolytic therapy should be used carefully, considering that such events can be self-limited, and that COVID-19 is also associated with thrombocytopenia and coagulopathy, with risk for developing widespread intravascular coagulation^26^. In addition, prophylaxis measures should be implemented during hospitalization and after discharge^40^.

### Perforation

A case report by De Nardi et al.^14^ described a patient with COVID-19 in whom ascending colon perforation evolved. An acute overdistension of the right colon, without mechanical obstruction, led to colon perforation. The pathophysiology of this event remains unclear; however, findings, including ACE2 protein and cell receptor for SARS-CoV-2, were observed in glandular cells of the gastric, duodenal, and rectal epithelia, which suggests a tropism of SARS-CoV-2 for cells of the gastrointestinal tract. Another hypothesis refers to the neuroinvasive potential of coronaviruses, which can generate an imbalance in the autonomic innervation of the colon, thus altering its motility^14^ ^,^ ^24^.

In another report, Ibrahim et al.^24^ described a case with extensive large bowel distension and perforation of the mid-transverse colon. The histopathology of the resected sample revealed fat necrosis, acute inflammation, reactive fibroblastic proliferation, and hemorrhage. However, the mesenteric vessels appeared to be pervious on imaging, suggesting that the etiology could be microthrombosis induced by SARS-CoV-2. It was hypothesized that it causes inflammation of vascular endothelial cells, leading to impaired microcirculatory function in various vascular beds.

In a case report by Dick et al.^25^, a patient with a typical presentation of COVID-19 and a positive RT-PCR test for SARS-CoV-2 was placed on mechanical ventilation and parenteral nutrition and began exhibiting significant abdominal distention. Aspiration of the abdominal contents, similar to that of food, raised suspicion for gastrointestinal perforation, which was later confirmed using the methylene blue test. The possible cause of the complication was an ulcer, and the hypothesis that ACE2, as a route of infection for the virus in the gastrointestinal tract, was proposed once again.

Nahas et al.^36^, as well as De Nardi et al.^14^, described a case of colonic perforation, this time in its descending portion. A 92-year-old patient underwent emergency surgery due to intestinal obstruction due to a rectal tumor, developing on the 10th postoperative day with symptoms of COVID-19, confirmed by swab oro and nasopharyngeal PCR, in addition to abdominal pain and oliguria. A punctiform perforation of the descending colon was identified and treated in exploratory laparotomy. After an unfavorable evolution of the condition and death, the anatomopathological analysis showed thrombosis of the local microcirculation, which the authors considered as a triggering factor for ischemia and perforation of the colon wall.^36^

### Acute pancreatitis

It is well known that ACE2 receptors can play a role in the pathogenesis of COVID-19, and that these transmembrane proteins are expressed more in the pancreas than in the lung. In addition, single cell RNA sequencing data indicated that ACE2 is expressed in exocrine glands and islets of the pancreas^2^ ^,^ ^5^ ^,^ ^9^ ^,^ ^23^ ^,^ ^30^ ^,^ ^42^ ^,^ ^44^.

Schepis et al.^42^ reported, for the first time, the quantitative detection of SARS-CoV-2 RNA in fluid collected from the pancreas, which supports the involvement of the gastrointestinal system-and the pancreas in particular-in COVID-19. However, it is unclear whether COVID-19-related acute pancreatitis is due to the direct cytopathic effect of local viral replication or indirectly by a harmful immune response precipitated by SARS-CoV-2 infection^2^ ^,^ ^5^ ^,^ ^9^ ^,^ ^30^ ^,^ ^42^ ^,^ ^44^. 

Liu et al.^30^ reported that approximately 1-2% of patients with non-severe and 17% with severe COVID-19 incurred pancreatic injury. These clinical data demonstrate that pancreatic injury can occur, especially in patients experiencing severe forms of the disease.

Therefore, gastrointestinal symptoms and abdominal pain in COVID-19 patients should be carefully evaluated. The measurement of amylase and lipase levels, and the use of imaging studies, such as abdominal and pelvic CT, can be crucial in these patients to enable early diagnosis and intervention and, consequently, better outcomes^5^ ^,^ ^23^ ^,^ ^27^.

### Pneumatosis intestinalis

SARS-CoV-2 uses the ACE2 receptor to enter cells and the serine protease TMPRSS2 for protein S priming. These two proteins are highly co-expressed, not only in type 2 alveolar cells, but also in enterocytes of the ileum and colon, suggesting that the virus is capable of invading the digestive tract. Fecal-oral transmission is a possible route of transmission of SARS-CoV-2, which can be detected in feces in approximately 50% of patients with COVID-19. However, there is no clear correlation between gastrointestinal symptoms and detectable virus in the stool. The pathogenesis of pneumatosis intestinalis is poorly understood. It is believed that the intestinal microbiota can be altered, and that pneumatosis intestinalis may result from intraluminal gas produced by the overgrowth of gas-forming bacteria, with subsequent diffusion of this gas in the submucosa. The gas can enter the intestinal wall due to the coexistence of increased intraluminal pressure, rupture of the mucosa, and increased permeability. In addition, the excessive production of hydrogen due to bacterial growth can lead to gas oversaturation, thereby overloading diffusion capacity in the bloodstream and, consequently, leading to the formation of cysts containing gas^34^.

## CONCLUSION

Considering the relationship between high expression levels of ACE2 in the gastrointestinal tract and its greater susceptibility to direct infection by SARS-CoV-2 is established, it is important to consider the various gastrointestinal manifestations and complications in these patients. The most important include gastrointestinal hemorrhage, thrombotic and ischemic events, perforation, pancreatitis and, less commonly, pneumatosis intestinalis. Therefore, when managing patients with known SARS-CoV-2 infection, or even initially without a diagnosis of COVID-19, all gastrointestinal manifestations should be considered, diagnosed, and treated early to prevent even more serious complications and death.
